# Antifungal Effects of Volatiles Produced by *Bacillus subtilis* Against *Alternaria solani* in Potato

**DOI:** 10.3389/fmicb.2020.01196

**Published:** 2020-06-17

**Authors:** Dai Zhang, Shuiqing Yu, Yiqing Yang, Jinglin Zhang, Dongmei Zhao, Yang Pan, Shasha Fan, Zhihui Yang, Jiehua Zhu

**Affiliations:** ^1^College of Plant Protection, Hebei Agricultural University, Baoding, China; ^2^Beijing Laboratory for Food Quality and Safety, Beijing Technology and Business University, Beijing, China

**Keywords:** antifungal activity, *Bacillus subtilis*, volatile organic compounds, airborne phytopathogens, *Alternaria solani*

## Abstract

Antifungal activities of plant-beneficial *Bacillus* have been widely studied in recent years. Numerous studies have studied the antifungal mechanisms of soluble non-volatile bioactive compounds such as lipopeptides and proteins produced by *Bacillus* against soil-borne diseases. However, the antagonistic mechanisms of volatile organic compounds (VOCs) from *Bacillus* against airborne phytopathogens are still largely unknown, and the function of *Alternaria solani* pathogenic genes has not been well identified. Here, we first isolated a *Bacillus* strain with strong antifungal activity and finally identified it as *B. subtilis* ZD01. Then, the antagonistic mechanisms of VOCs produced by strain ZD01, against *A. solani*, an airborne fungal pathogen that can cause early blight diseases of potato, were studied. We showed that VOCs produced by strain ZD01 can reduce the colony size and mycelial penetration and can cause serious morphological changes of *A. solani*. Scanning electron microscope (SEM) observation showed that VOCs released by ZD01 could cause more flaccid and gapped hyphae of *A. solani*. Also, we found that VOCs produced by ZD01 can inhibit the conidia germination and reduce the lesion areas and number of *A. solani in vivo* significantly. Meanwhile, based on gas chromatography/mass spectrometry (GC/MS) analysis, 29 volatile compounds produced by strain ZD01 were identified. Out of 29 identified VOCs, 9 VOCs showed complete growth inhibition activities against *A. solani*. Moreover, we identified two virulence-associated genes (*slt2* and *sod*) in *A. solani*. *slt2* is a key gene that regulates the mycelial growth, penetration, sporulation, and virulence *in vivo* in *A. solani*. In addition, *sod* plays a significant role in the SOD synthetic pathway in *A. solani*. Results from qRT-PCR showed that the transcriptional expression of these two genes was down-regulated after being treated by VOCs produced by ZD01. These results are useful for a better understanding of the biocontrol mechanism of *Bacillus* and offer a potential method for potato early blight disease control.

## Introduction

*Alternaria solani* is a kind of fungal pathogen that can cause early blight disease of tomato, potato, tobacco, and many other vegetables and crops, and lead to huge losses in agricultural production. Potato is one of the most important crops in the world. Early blight disease on potato can cause up to 80% of annual yield losses in some regions of the world (Morgan et al., 2002; Pasche et al., 2004; Peters et al., 2008). Nowadays, chemical fungicides are the most effective agents to control early blight. However, the indiscriminate using and even abuse of chemical fungicides have already caused the appearance of resistant pathogens, which will further threaten the food safety and human health. Due to this, there is a rising interest to seek for alternative antifungal agents for plant disease control.

*Bacillus* species are known for their capacity to produce a great variety of antifungal compounds to suppress or kill fungal pathogens (Chaurasia et al., 2005). Among them, non-ribosomal cyclic lipopeptides (Nakano et al., 1988; Munimbazi and Bullerman, 1998; Kim et al., 2004; Timmusk et al., 2005) are the most well-studied ones. However, these non-volatile antibiotics cannot spread over long distances. In recent years, volatile organic compounds (VOCs) produced by *Bacillus* have been evaluated as a new approach for plant fungal disease control. Due to their capability of diffusing between the soil particles and spreading into the atmosphere over very large distances from their original application point, VOCs can exert their inhibitory activity without direct or physical contact between the VOC-producing microorganisms and the target pathogens (Minerdi et al., 2009; Heydari and Pessarakli, 2010). Their strong antifungal activities, along with the characteristic of harmless to both environment and human beings, make VOCs a promising and sustainable agent to replace fungicides for plant pathogen control in the future (Fialho et al., 2010; Parafati et al., 2017; Tilocca et al., 2020).

In many previous studies, VOCs emitted by *Bacillus* species were shown to be potential antifungal agent against many soil-borne pathogens. For instance, VOCs produced by *B. amyloliquefaciens* NJN-6 could hinder growth and spore germination of the pathogenic *Fusarium oxysporum* f. sp. *cubense* causing fusarium wilt on banana (Yuan et al., 2012). Moreover, numerous identified volatiles released by *Bacillus* have demonstrated to inhibit fungal growth, including dimethyl disulfide, 1-undecene, benzaldehyde, benzothiazole, dimethyl trisulfide, cyclohexanol, decanal, 2-ethyl-1-hexanol (Kai et al., 2009). For instance, co-cultured with 2-nonone and 2-heptanone released by *B. amyloliquefaciens*, the mycelia of *F. oxysporum* f. sp. *niveum* stopped growth completely (Wu et al., 2019).

However, limited knowledge is known about bacterial VOCs controlling airborne plant fungal pathogens, especially *A. solani*. In several recent studies, VOCs produced by *Bacillus* species have been identified to inhibit the airborne pathogens, mainly as *Botrytis cinerea*, the causal agent of tomato gray mold. Two *Bacillus velezensis* strains 5YN8 and DSN012 could suppress the growth and spore formation of *B. cinerea* by releasing numbers of VOCs (Jiang et al., 2018). The VOCs of *B. velezensis* ZSY-1 strain exhibit significant antifungal activity against *B. cinerea* (Gao et al., 2017). The mixed volatiles produced by *B. atrophaeus* CAB-1 strains, mainly composed of hexadecane, 2,3-dimethoxybenzamide, and oanisaldehyde, resulted in an effective inhibition of *B. cinerea* (Zhang et al., 2013).

In this study, *B. subtilis* ZD01, an effective antifungal strain, was isolated and identified from potato rhizosphere, and the antifungal effects of VOCs produced by it on *A. solani* were investigated by both *in vivo* and *in vitro* experiments. We demonstrated that VOCs of *B. subtilis* have great potential to be used as a biologically synthesized fungicide in the agricultural field.

## Results

### *B. subtilis* ZD01 Showed Strong Inhibitory Activity Against *A. solani*

**FIGURE 1:**
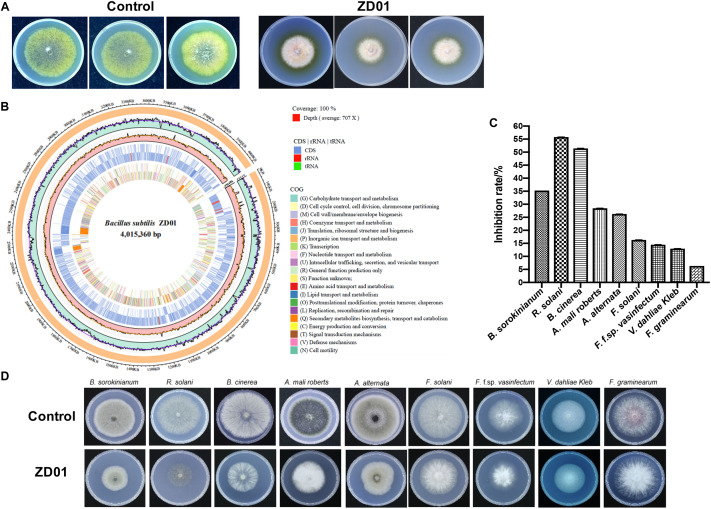
Isolation and identification of ZD01 volatiles for *A. solani* control. **(A)** Antagonistic activity of ZD01 volatiles against *A. solani*. **(B)** The circularized genome map of *Bacillus subtilis* ZD01. **(C,D)** Antagonistic activity of VOCs produced by ZD01 against nine plant pathogens, including *Bipolaris sorokinianum*, *Rhizoctonia solani*, *Botrytis cinerea*, *Alternaria mali roberts*, *Alternaria alternata*, *Fusarium solani*, *Fusarium oxysporum* f. sp. v*asinfectum*, *Verticillium dahliae Kleb*, and *Fusarium graminearum.* Control represents *A. solani* without treatment of ZD01 VOCs; ZD01 represents *A. solani* with treatment of ZD01 VOCs.

A total of 103 isolated *Bacillus* strains were obtained from rhizosphere of potato in Shandong and Hebei Province in China. Among them, 34 isolates showed a certain inhibition effect on *A. solani* (Supplementary Table S1). Notably, strain ZD01 showed the strongest antagonistic activity against *A. solani* (Supplementary Table S1). Then, ZD01 were tested for inhibitory activity mediated by volatile against *A. solani* using face-to-face Petri dish method (Gao et al., 2017). The result showed that the growth rates of *A. solani* mycelia were reduced by 50.1 ± 2.1% in the presence of volatiles released by ZD01 (Figure 1A). Figures 1C,D showed that a broad range of pathogens were also inhibited by the volatiles released from strain ZD01.

Strain ZD01 was then identified by 16s rRNA and complete genome sequencing. We performed BLAST search using the DNA sequence of the16S rRNA gene of ZD01 as a query against the NCBI GenBank database. Furthermore, we sequenced the complete genome of ZD01 by Pacbio RSII. After that, a single circularized chromosome of 4,015,360 bp in length with a GC content of 43.71% from ZD01 was obtained (Figure 1B, CP046448). According to the BLAST and complete genome sequencing results, we found that strain ZD01 showed extremely high similarity (identity 99%, *E*-value = 0) to *B. subtilis*. We finally classified the strain ZD01 as *B. subtilis*.

### VOCs Produced by ZD01 Changed the Mycelia Morphology and Inhibited the Conidia Germination of *A. solani*

**FIGURE 2:**
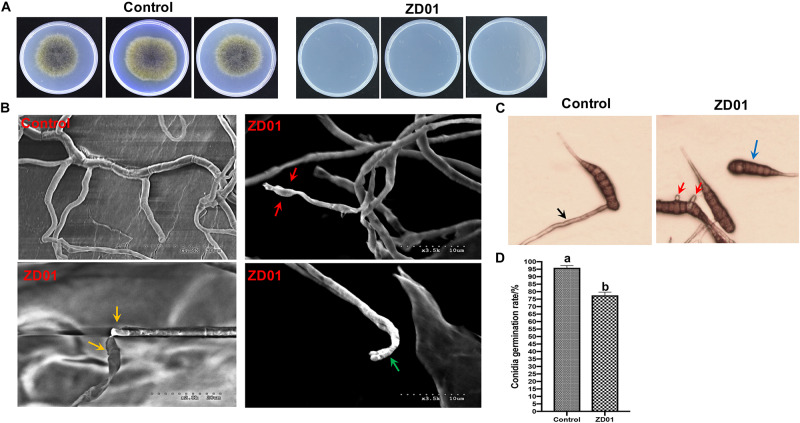
Effects of strain ZD01 volatiles on mycelia penetration and morphology as well as conidia germination of *A. solani*. **(A)** Mycelia penetration inhibition by ZD01 volatiles. **(B)** Scanning electron micrographs of *A. solani* co-cultured with ZD01 volatiles. **(C,D)** Reduction of conidia germination of *A. solani* with the treatment of ZD01 volatiles. Control represents *A. solani* without treatment of ZD01 VOCs; ZD01 represents *A. solani* with treatment of ZD01 VOCs. Data presented are the mean ± s.d. (*n* = 3). The same letter on the bars for each column indicates no significant difference according to a LSD test at *P* = 0.05.

Inhibition of fungal mycelia growth and spore germination is conventional mechanism of biocontrol agents for disease controlling. So, the suppression of VOCs produced by ZD01 on mycelia growth and conidia germination were evaluated. The result showed that the inhibition rate of *A. solani* growth by VOCs was 50.1 ± 2.1% compared with the control after 6 days, suggesting that the VOCs from ZD01 showed a strong inhibitory activity on fungal mycelia growth. Moreover, with the treatment of VOCs produced by *B. subtilis* ZD01, the mycelia of *A. solani* became denser and thicker, and the color of the colony turned into white (Figure 1A). In addition, strong decrease in mycelial penetration was observed after treatment. Mycelia in control groups were able to penetrate cellophane sheets during the cellophane penetration assay with the colony diameter of 5.5 ± 1.6 cm. However, the *A. solani* colony co-cultured with VOCs produced by ZD01 did not grow after the cellophane was removed, which indicated that the penetration ability of mycelia lost completely (Figure 2A).

A scanning electron microscope (SEM) was used to investigate detailed morphological changes of *A. solani* hyphae caused by VOCs. Morphological changes in *A. solani* cells are shown in Figure 2B. Regular length and intact cell walls with uniform composition and structure were present in the hyphae of *A. solani* in the control group (Figure 2B). However, hyphae treated with VOCs from ZD01 exhibited substantial structural destruction (Figure 2B). In detail, some of the hyphae became expanded, and the formation of empty segments was presented (red arrows, Figure 2B). Treated mycelium appeared with a more flaccid hyphae, and the surface of the cell walls became uneven (yellow arrows, Figure 2B). Also, thin or gapped structures presenting a retracted protoplasm were seen in Figure 2B (green arrows). The broken structures might lead to the leakage of cytoplasmic components. Thus, volatiles produced by ZD01 could change the morphology of *A. solani* seriously, and decompose the cell walls and membrane.

Spore germination plays a crucial role during the infection of fungal pathogens, so the conidia germination suppression capacity of VOCs produced by *B. subtilis* ZD01 was evaluated. As shown in Figures 2C,D, the *B. subtilis* ZD01 could inhibit the germination of *A. solani* conidia significantly (*P* < 0.05) by releasing some VOCs compared with the control group. In the control group, conidia began to germinate and germ tube formed. Then, the germ tubes formed regular hyphae (black arrow, Figure 2C), which can directly penetrate the host epidermal cell junctions and formed infection hyphae on leaf. However, some conidia treated with VOCs from ZD01 could not germinate completely (blue arrow, Figure 2C), and some conidia formed irregular germ tubes, which were much shorter than those in the control group (red arrows, Figure 2C). The inhibition rate of VOCs from ZD01 on conidia germination was 19.2 ± 2.1% (Figure 2D).

### VOCs Produced by *B. subtilis* ZD01 Reduced the Symptoms of Early Blight Disease on Potato Leaf

**FIGURE 3:**
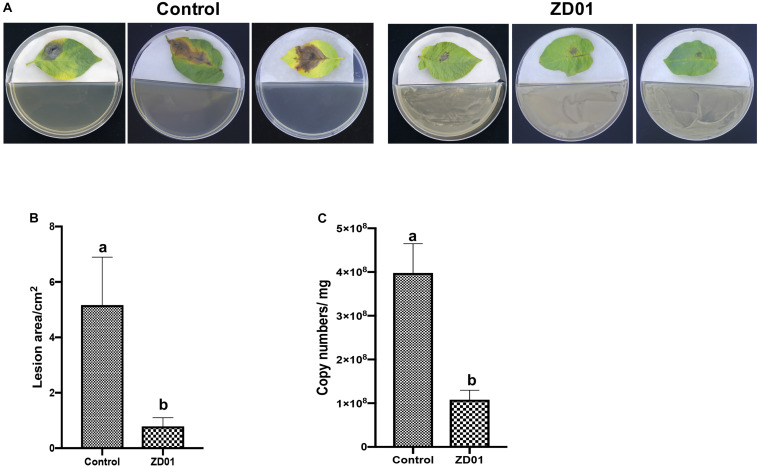
Antagonistic effects of VOCs produced by strain ZD01 against *A. solani* on potato leaf. **(A)** Effect of VOCs produced by ZD01 on development of early blight symptoms on Potato leaf. **(B)** Lesion areas of potato leaf with or without the treatment by ZD01 volatiles. **(C)** *A. solani* copy numbers per leaf were detected in potato leaf. Control represents *A. solani* without treatment of ZD01 VOCs; ZD01 represents *A. solani* with treatment of ZD01 VOCs. Data presented are the mean ± s.d. (*n* = 3). The same letter on the bars for each column indicates no significant difference according to a LSD test at *P* = 0.05.

The development and expansion of disease symptom induced by *A. solani* was inhibited effectively by VOCs produced by strain ZD01 in *in vivo* leaf test. As shown in Figure 3A, the lesion areas on potato leaves (cultivar Helan 15) inoculated by *A. solani* were significantly reduced (*P* < 0.05) after co-cultivation with ZD01 volatiles by divided plate method. For leaves in the control group with no treatment of ZD01 volatiles, the lesion areas extended to 5.2 ± 1.7 cm^2^ after 5–7 days incubation at 25°C, whereas for the leaves exposed to volatiles from ZD01, the lesion areas were limited to 0.8 ± 0.3 cm^2^ (Figure 3B). These results corresponded to relative pathogen copy numbers per milligram leaf of 3.98 ± 0.67 × 10^8^ and 1.08 ± 0.22 × 10^8^ for control and treatment, respectively (Figure 3C).

### Identification and Antifungal Activity of VOCs Produced by *B. subtilis* ZD01

**FIGURE 4:**
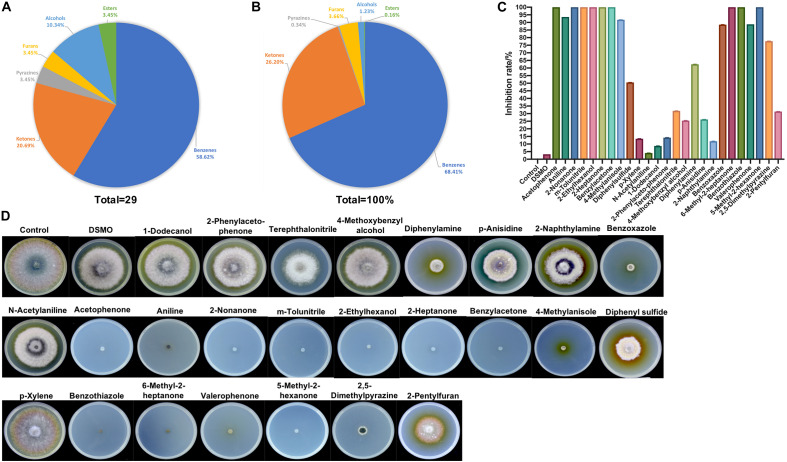
Identification of ZD01 volatiles and inhibition effects of selected VOCs on *A. solani*. **(A)** Classification of VOCs produced by ZD01. **(B)** Peak area of six identified classes of VOCs from ZD01. **(C)** Inhibition rate of 25 identified VOCs against *A. solani*. **(D)** Colony diameter of *A. solani* co-cultured with 25 identified VOCs. Control represents *A. solani* without treatment of pure identified VOCs.

The VOCs produced by strain ZD01 were analyzed by solid-phase microextraction–gas chromatography/mass spectrometry (SPME-GC/MS). In total, 29 VOCs were identified from ZD01, including 6 ketones, 17 aromatic compounds, 1 furan, 1 pyrazine, 3 alcohols, and 1 ester (Figures 4A,B and Table 1).

**TABLE 1:** Volatile compounds produced by *B. subtilis* ZD01.

Chemicals	CAS	Retaining time (min)	Similarity degree	Retention index	Peak area ratio (%)
5-methyl-2-hexanone	110-12-3	7.57	94	853	1.99
p-xylene	106-42-3	7.88	90	863	0.78
2-heptanone	110-43-0	8.68	90	888	1.05
2,5-dimethylpyrazine	123-32-0	9.34	94	908	0.34
6-methyl-2-heptanone	928-68-7	10.91	97	954	8.88
5-methyl-2-heptanone	18217-12-4	11.2	90	962	12.81
Aniline	62-53-3	11.55	97	973	31.39
2-pentylfuran	3777-69-3	12.12	86	990	3.66
Benzoxazole	273-53-0	12.88	85	1013	0.08
4-methylanisole Anisole	104-93-8	12.98	80	1016	0.17
2-ethylhexanol	104-76-7	13.38	90	1028	0.42
2-Nonanone	821-55-6	14.2	90	1054	1.30
Acetophenone	98-86-2	14.47	98	1062	18.54
Allypropyl ether	1471-03-0	15.37	90	1090	0.16
m-tolunitrile	620-22-4	15.65	92	1098	0.56
Acrylophee	768-03-6	17.26	91	1151	2.18
Propiophenone	93-55-0	17.57	92	1161	1.63
p-anisidine	104-94-9	19.16	91	1215	0.55
Benzothiazole	95-16-9	19.31	98	1220	3.01
Benzylacetone	2550-26-7	19.89	92	1240	0.17
Terephthalonitrile	623-26-7	20.42	86	1259	0.11
4-methoxybenzyl alcohol	105-13-5	20.99	86	1279	0.62
Valerophenone	1009-14-9	23.03	92	1354	2.21
*N*-acetylaniline	103-84-4	23.57	94	1374	0.28
2-naphthylamine	91-59-8	25.79	91	1459	3.53
1-dodecanol	112-53-8	26	94	1467	0.19
Diphenyl sulfide	139-66-2	28.83	92	1587	0.43
Diphenylamine	122-39-4	29.49	90	1620	0.69
2-Phenylacetophenone	451-40-1	31.34	94	1731	2.28

To determine the antagonistic effect of VOCs produced by ZD01, 25 of the identified VOCs were tested using face-to-face plate method (Gong et al., 2015). Among the 25 volatile chemicals, nine chemicals including acetophenone, 2-nonanone, m-tolunitrile, 2-ethylhexanol, 2-heptanone, benzylacetone, 6-methyl-2-heptanone, benzothiazole, and 5-methyl-2-hexanone completely inhibited the growth of *A. solani*. Aniline, 4-methylanisole, benzoxazole, valerophenone, and 2,5-dimethylpyrazine showed strong antagonistic effect, and their inhibition rates against *A. solani* were 93.6 ± 0.3, 91.7 ± 1.6, 88.5 ± 4.6, 88.8 ± 0.5, and 77.7 ± 6.0%, respectively. The remaining compounds showed weak or no inhibitory activity (Figures 4C,D). Among these active VOCs, acetophenone, 6-methyl-2-heptanone, and aniline have larger peak areas than others, with 18.5, 8.9, and 31.4%, respectively (Table 1), which indicated that these compounds are potential agents for controlling potato early blight.

### *slt2* and *sod* Are Virulence-Associated Genes in *A. solani*

Identification of virulence-associated genes is important to reveal the pathogenic mechanisms and biological control approaches of fungal pathogens. The complete genome sequence of *A. solani* HWC-168 has been sequenced and analyzed in our previous study (Zhang et al., 2018). We compared the whole genome sequence of HWC-168 with its well-studied *Saccharomyces cerevisiae* (EF058927.1) and close relative *A. alternate* (GQ414510.1). After that, two typical pathogenic genes (*slt2* and *sod*) were found in the genome of HWC-168. Then, the functions of these two genes were determined through gene knockout and phenotype verification.

**FIGURE 5:**
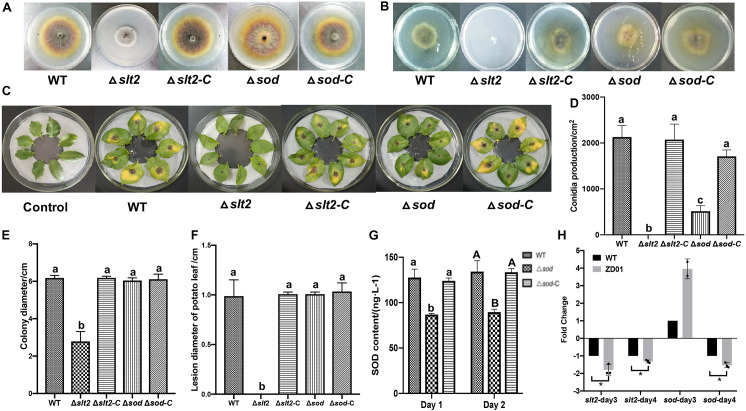
*slt2* and *sod* are key virulence related genes in *A. solani.* **(A)** Colony morphology of wild-type *A. solani* (WT), mutants (Δ*slt2* and Δ*sod*) and complementation strains (Δ*slt2-*C and Δ*sod-*C). **(B)** Mycelia penetration ability of wild-type *A. solani* (WT), mutants (Δ*slt2* and Δ*sod*), and complementation strains (Δ*slt2-*C and Δ*sod-*C). **(C)** Symptoms of early blight disease on potato leaf caused by sterile water (Control), wild-type *A. solani* (WT), mutants (Δ*slt2* and Δ*sod*), and complementation strains (Δ*slt2-*C and Δ*sod-*C). **(D,E)** Conidia production and colony diameter of wild-type *A. solani* (WT), mutants (Δ*slt2* and Δ*sod*), and complementation strains (Δ*slt2*-C and Δ*sod*-C). **(F)** Lesion diameter early blight disease on potato leaf caused by wild-type *A. solani* (WT), mutants (Δ*slt2* and Δ*sod*), and complementation strains (Δ*slt2-*C and Δ*sod-*C). **(G)** SOD contents in wild-type *A. solani* (WT), mutants (Δ*slt2* and Δ*sod*), and complementation strains (Δ*slt2-*C and Δ*sod-*C). **(H)** Transcriptional expression profiles of *slt2* and *sod* after co-culture with ZD01 VOCs for 3 and 4 days. WT represents *A. solani* without treatment of ZD01 VOCs; ZD01 represents *A. solani* with treatment of ZD01 VOCs. Data presented are the mean ± s.d. (*n* = 3). The same letter on the bars for each column indicates no significant difference according to a LSD test at *P* = 0.05.

To determine whether *slt2* and *sod* affects the pathogenicity of *A. solani*, we compared the deletion mutants with the wild-type strain and complementation strains in mycelia growth, sporulation, and virulence by *in vivo* potato leaf tests. Compared with the wild-type strain and complementation strains, the mycelium of *slt2* mutant was significantly denser, and the color of colony was gray-white without pigments (Figure 5A). The colony diameter of *slt2* deletion mutants was 2.79 ± 0.52 cm, while that of wild-type strain and complementation strains were 6.18 ± 0.14 and 6.18 ± 0.08 cm, respectively (Figure 5E). No obvious changes of the colony morphology and diameter of *sod* deletion mutant were observed (Figures 5A,E), while the colony diameter of Δ*sod* and its complementation strain (Δ*sod-*C) were 6.04 ± 0.14 and 6.11 ± 0.27 cm, respectively. These results suggested that *slt2* is the key gene involved in the regulation of mycelium growth and development. Also, we found that the *slt2* gene was critical for the penetration ability of *A. solani*. As shown in Figure 5B, the wild-type and complemented strains of *slt2* but not the mutants were able to penetrate cellophane sheets in the cellophane penetration assay.

We next tested whether the *slt2* and *sod* genes are also responsible for the sporulation of *A. solani*. The results showed that *slt2* mutant lost the capacity of conidia production and the yield of conidia of *sod* deletion mutant per area was 514 ± 149/cm^2^, while the wild-type strain was 2131 ± 301/cm^2^ (Figure 5D). Compared with the wild-type strain, the sporulation yield of *slt2* and *sod* deletion mutants per area decreased significantly (*P* < 0.05). Through the complementation strain, the yield of sporulation increased to 2073 ± 415/cm^2^ and 1709 ± 171/cm^2^, respectively. The sporulation ability of the *slt2* deletion mutant was completely lost, and the sporulation ability of the *sod* deletion mutants was weakened compared to that of the wild-type strain, suggesting that *slt2* is a key gene regulating the sporulation in *A. solani*. Meanwhile, a reduction of Δ*sod* mutant in superoxide dismutase (SOD) content was detected. The content of SOD in mutant was 86.8 ± 1.4 and 89.6 ± 3.2 ng/L, respectively, after 1 and 2 days incubation as compared to the wild-type HWC-168, which was 127.7 ± 9.2 and 134.2 ± 12.0 ng/L, respectively (Figure 5G). In addition, SOD contents in complementation strains were similar to those of the wild type (124.0 ± 3.2 ng/L, 133.5 ± 3.9 ng/L). These results suggested that *sod* has a significant role in the SOD synthetic pathway in *A. solani*.

In *in vivo* tests, potato leaves inoculated with wild-type HWC-168, and complementation strains of Δ*slt2-C* and Δ*sod-C* showed obvious lesions and yellow halos (Figure 5C). The lesion diameter extended to 0.99 ± 0.16, 1.01 ± 0.02, and 1.03 ± 0.09 cm after 7 days incubation at 25°C, whereas for the leaves inoculated with Δ*slt2* and Δ*sod* mutants, the lesion diameters were limited to 0.00 ± 0.00 cm and 0.73 ± 0.07 cm, respectively (Figure 5F). The result showed that the deletion of *slt2* and *sod* can significantly reduce the pathogenicity of *A. solani* (*P* < 0.05).

### *B. subtilis* ZD01 Volatiles Down-Regulated the Expression of Virulence-Associated Genes in *A. solani*

The transcriptional expression profiles of *slt2* and *sod* under condition of co-culture with ZD01 volatiles were investigated by real-time RT-PCR. The results showed that after *A. solani* strain HWC-168 was exposed to volatiles emitted by ZD01 for 3 days, the transcriptional expression of *sod* was strongly induced (∼2.45-fold) compared with the control group and then repressed (∼0.61-fold) after 4 days (Figure 5H). The expression of *slt2* was strongly repressed (∼0.83- and 0.40-fold) in the presence of VOCs after 3- and 4-day co-culture (Figure 5H). The down-regulated expression of *slt2* was consistent with the virulence reduction in *A. solani*.

## Discussion

**FIGURE 6:**
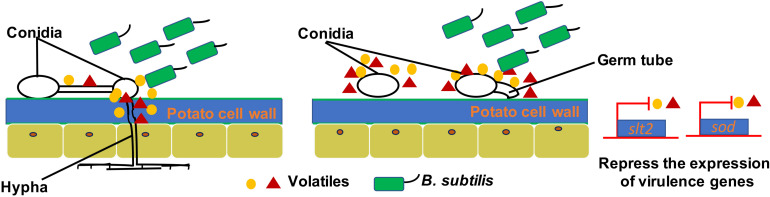
A model for the mode of action of VOCs produced by ZD01 against *A. solani*. Volatiles produced by ZD01 mediate the conidia germination and mycelium penetration of *A. solani*. Acetophenone, 6-methyl-2-heptanone, and aniline biosynthesized by ZD01 is the major antifungal compounds against *A. solani*. These volatiles produced by ZD01 target the conidia and mycelium, which subsequently leads to suppression of fungal growth, mycelium penetration, conidia germination, and pathogenicity of *A. solani* as well as virulence gene expression.

Many plant-beneficial *Bacillus* species exhibit their biocontrol capacity to plant pathogens through non-volatile antibiotic production, nutrients, and niche competitions, as well as induction of plant systemic resistance (Pliego et al., 2008; Lemfack et al., 2014; Farag et al., 2017; Xie et al., 2018). However, limited knowledge is known about the antifungal mechanisms of volatiles produced by *Bacillus* strains. Most studies just focus on mycelia morphology and penetration and spore germination at a cellular level. However, the molecular mechanisms of antifungal activity have not been revealed. For example, transmission electron microscopy observation of fumigated and untreated *B. cinerea* showed excessive vesication or thickened cell walls in exposed conidia and increased strong retraction of plasma membrane in exposed hyphae (Li et al., 2012). The VOCs of *B. velezensis* 5YN8 can suppress the mycelium growth and conidia formation of *B. cinerea* BC1301 (Jiang et al., 2018). In this study, the insights into the mechanisms of *B. subtilis* ZD01 volatiles against *A. solani* showed similar biocontrol strategies. The hyphae penetration, conidia germination, and virulence of *A. solani* were significantly reduced when treated with VOCs produced by ZD01 (*P* < 0.05). Scanning electron microscopy showed thin, inward or gapped structures and altered surface morphology in the majority of *A. solani* cells after co-culture with strain ZD01 volatiles. Meanwhile, *A. solani* cells exposed to VOCs produced by strain ZD01 formed swollen part of hyphae with defective ability, leading to aborted invasion to the plant barrier. These results were consistent with the reduced pathogenicity *in vivo*. All of these findings indicated that the mode of action of volatiles can be explained, at least in part, by their activities that lead to the functional degradation through the damage of mycelium structure, killing *A. solani* cells partially, and the reduction of spore germination (Figure 6).

All of the active volatiles produced by bacteria so far can be grouped into alcohols, ketones, aldehydes, alkenes, alkynes, benzenes, esters, terpenoids, heterocycles, and sulfur-containing compounds (Fernando et al., 2005; Corcuff et al., 2011; Lemfack et al., 2014). Among nine identified chemicals with strong antifungal effects, the antifungal effects of 6-methyl-2-heptanone, acetophenone, 2-pentylfuran, 2,5-dimethyl pyrazine, and benzothiazole have also been analyzed. 6-Methyl-2-heptanone produced by *B. subtilis* completely inhibited mycelial growth of *F. oxysporum* f. sp. *lactucae* (Liu et al., 2018). Acetophenone released by *Streptomyces globisporus* and *Paenibacillus polymyxa* can inhibit *Penicillium italicum* and *F. oxysporum* growth, respectively (Li et al., 2010; Raza et al., 2015). The multiple functions of 2-pentylfuran have also been identified. 2-Pentylfuran isolated from the volatile products of bacterial strains presented strong inhibition on both mycelial growth and conidia germination of *F. oxysporum* (Wu et al., 2015; Liu et al., 2018). 2-Pentylfuran fumigated from *Bacillus megaterium* strain XTBG34 could significantly promote plant growth of *Arabidopsis thaliana* (Zou et al., 2010). Moreover, 2,5-dimethylpyrazine can significantly inhibit growth of several plant pathogens such as *Magnaporthe oryzae*, *Phytophthora capsici*, and *A. solani* (Munjal et al., 2016; Gao et al., 2017), and it can also be used as a food additive at low concentration (Müller and Rappert, 2010). Benzothiazole produced by *Bacillus* species can inhibit growth of *F. oxysporum* (Raza et al., 2015), and it can also be used to produce riluzole and pramipexole (Powell, 2015; Faridbod et al., 2016).

In this study, benzenes and ketones are the most abundant volatiles produced by strain ZD01. The aniline and acetophenone, which belonged to benzene compounds, could completely inhibit the growth of *A. solani* at certain concentrations and also exhibited high yield, as indicated by the peak areas of the GCs, so it was likely that they are main VOCs that play key roles during the antifungal process. For the VOCs that belong to ketones, 2-nonanone, 5-methyl-2-hexone, and 2-heptanone exhibited 100% inhibition of *A. solani* under certain concentrations when we did *in vitro* inhibition test, but their yields were relatively low among VOCs produced by ZD01, as indicated by GC-MS. However, 6-methyl-2-heptanone exhibited large peak areas on the GCs, and it did show strong antifungal effects against *A. solani* with 100% inhibition. Considering both contents and antifungal effects, aniline, acetophenone, and 6-methyl-2-heptanone might be considered active antifungal compounds.

Based on whole genome sequences and annotation of *A. solani* strain HWC-168 (Zhang et al., 2018), two virulence-related genes *slt2* and *sod* were predicted. In some fungal pathogens, like *Colletotrichum lagenarium*, *B. cinerea*, and *Mycosphaerella graminicola*, the role of virulence-related gene *slt2* has also been well studied, involved in the maintenance of cell-wall integrity and various aspects of saprotrophic and pathogenic growth (Kojima et al., 2002; Mehrabi et al., 2006; Rui and Hahn, 2007). In *B. cinerea*, a mutant defective in the *slt2* homolog *mp3* was defective in penetration and non-pathogenic infection (Rui and Hahn, 2007). Meanwhile, the biological role of *sod* gene has been extensively investigated in the model organism *S. cerevisiae.* However, the roles of these two genes in *A. solani* are still unclear. Here, we present the functions of *slt2* and *sod* in *A. solani* identified by knock-out mutant construction and phenotypical characterization. We found that *slt2* is a key gene involved in mycelial growth, hyphae penetration, and sporulation of *A. solani*, which could further affect its pathogenicity. Meanwhile, we also found that *sod* is responsible for the synthesis of SOD in *A. solani* and its deletion mutant can reduce virulence of *A. solani* to some extent. Furthermore, we found that VOCs produced by *B. subtilis* ZD01 can decrease the transcriptional expression of these two genes. The down-regulated expression of *slt2* was consistent with the reduced virulence in *A. solani*. However, the expression of *sod* was induced co-cultured with VOCs produced by strain ZD01 after 3 days. *A. solani* responds to and resists the biocontrol agent, *B. subtilis* VOCs, resulting to produce the high level of reactive oxygen species (ROS). SOD, encoded by *sod*, is involved in scavenging the high level of ROS into molecular oxygen and hydrogen peroxide. Then, with the accumulation of VOCs emitted by strain ZD01, the pathogenicity of *sod* decreased along with the increasing VOCs. In addition, VOC composition of a given species is highly dynamic over time, resulting in a changing composition of the produced VOCs depending on the age of the VOC-producing species (Wang et al., 2013). That would be the reason for the induced *sod* response after 3 days and repression after 4 days. These results reveal the molecular mechanism by which VOCs antagonize *A. solani*.

This study shed light on the interaction mechanisms between *A. solani* and VOCs produced by *B. subtilis*, and a potential biocontrol method for potato early blight disease caused by *A. solani*.

## Experimental Procedures

### Strains and Culture Conditions

Soil samples were collected from four different fields (Chengde, Hebei; Zhang Jiakou, Hebei; Qinhuangdao, Hebei; Tengzhou, Shandong) which had serious potato early blight disease by a five-spot sampling method (Chen et al., 2018) in China. Briefly, five soil samples that were 0–20 cm away from infected potato plants (Helan 15) were collected and then pooled as one sample. After that, the dilution plating method with miner modification was used for spore-forming bacteria isolation (López-Berges et al., 2010). In brief, soil sample was homogenized and placed in a sterilized beaker. Macerated samples were heated in a water bath for 15 min at 80°C to kill non-spore-forming bacteria and vegetative cells of spore-forming bacteria. The resulting supernatants were serially diluted in sterile 0.85% NaCl solution and plated onto LB agar plates. Plates were incubated at 37°C for 1 day.

Collected strains were tested for antagonistic activity. Briefly, bacterial antagonistic activities toward *A. solani* were tested by dual-culture assay on PDA (Hu et al., 2014), which support the vegetative growth of either bacteria or fungi. The bacterial strains (5 μl, 1 × 10^8^ CFU/ml) were inoculated 2 cm away from *A. solani*. Four strains were spotted in each dish, and the blank control without bacterial strains was set. All isolates were tested in triplicate, and their inhibition zones were measured after 7 days of dual culture at 25°C.

Isolated bacterial strains were cultured in LB medium at 37°C. Strain ZD01 was then identified by 16S rRNA gene analysis and whole genome sequencing (CP046448). The genome of ZD01 was sequenced using the PacBio RSII sequencing platform (Pacific Biomarkers, CA, United States). All the strains used in this study are listed in Supplementary Table S2.

### Antagonistic Assay of VOCs Against Fungal Pathogens

By following the methods of Gong et al. (2015), two Petri dishes were placed face to face. The bottom Petri dish contained LB agar (1.5%, wt/vol), which was inoculated with ZD01 (200 μl, 1 × 10^8^ CFU/ml), or pure identified VOCs (100 μl; 1 g/ml) were added. All the commercial solid VOCs were dissolved in dimethyl sulfoxide (DMSO). Purchase information of all the used reagents is listed in Supplementary Table S3. The top Petri dish contained 1.5% of potato dextrose agar (PDA). For the growth inhibition test, plugs (5 mm in diameter) of plant pathogenic fungi were placed onto the center of PDA plates. All the used fungal pathogens are listed in Supplementary Table S2. For the conidia germination inhibition test, 100 μl of conidia solution (10^5^ CFU/ml) was added. The top and bottom Petri dishes were sealed together with parafilm and incubated at 25°C. After 4–7 days, the diameters of the fungal colonies were measured. Inhibition of *A. solani* conidia germination by VOCs was determined after 8 h of incubation. LB plates without bacterial inoculum were used as control. Inhibition rate of mycelium growth and conidia germination was calculated by the following formulas:

Inhibitionrateofmyceliumgrowth(%)=(thediameterofcontrol-thediameteroftreatmentgroup)/the⁢diameter⁢of⁢control×100%

Inhibitionrateofconidiagermination(%)=(theconidiagerminationofcontrol-theconidiagermination

oftreatmentgroup)/theconidiagerminationofcontrol×100%

### Penetrability Assays

The penetration ability of each *A. solani* strain was examined on grown PDA plates covered with cellophane membranes, as described previously (López-Berges et al., 2010). Briefly, each strain was grown on PDA plates covered with a cellophane membrane. For the VOC treatment groups, plugs (5 mm in diameter) of wild-type *A. solani* were spotted onto the center of PDA plate with a cellophane. Then, the wild-type strains were co-cultured with VOCs produced by strain ZD01 in LB plate (200 μl, 10^8^ CFU/ml) for 3 days at 25°C using the face-to-face Petri dishes. For another 3 days, the wild-type strain was grown without VOC treatment and the cellophane membrane on the PDA plate was removed. Then, the penetrability halo was examined. LB plates without bacterial inoculum were used as control. For mutants and complementation strains, plugs (5 mm in diameter) of wild type, mutants of Δ*slt2* and Δ*sod*, and complementation strains of *A. solani* were spotted onto the center of the PDA plate covered with the cellophane, respectively. After 3 days of incubation at 25°C, the cellophane membrane with the colony was removed from each plate. Each strain was grown for another 3 days at 25°C. Then, penetrated mycelial growth on each plate was examined after incubation. The experiment was repeated three times.

### Scanning Electron Microscopy

The mycelia morphologies of *A. solani* in control groups or those treated with VOCs released by ZD01 were visualized by SEM. To observe structural changes on *A. solani*, the wild-type strains were co-cultured with VOCs produced by strain ZD01 for 6 days at 25°C using the face-to-face Petri dishes. Mycelia of each group were harvested and fixed in 2% glutaraldehyde at 4°C and then dehydrated with gradient ethanol solutions (30, 50, 80, 90, and 100%). After that, ethanol was replaced by 100% tertiary butyl ethanol. Cells were then freeze-dried, coated with gold, and imaged using a Hitachi S-3500N field emission SEM (Hitachi, Tokyo, Japan). The experiment was repeated three times.

### *In vivo* Antagonistic Activity of VOCs Produced by ZD01

Strain ZD01 was inoculated in 2 ml of LB broth and grown overnight. 1% of overnight culture was re-inoculated into 50 ml of fresh LB broth and incubated at 37°C under the shaking condition of 200 rpm for 12 h. Subsequently, 200 μl of cell-free supernatant was transferred into one compartment of the divided Petri dish with 1.5% of LB agar and spread out. One piece of fresh potato leaf (Helan 15) was placed onto the other compartment with 0.5% water agar containing 10 μg/ml tetracycline hydrochloride and 20 μl of *A. solani* conidia suspension (10^5^ CFU/ml) was inoculated onto the center of the leaf. LB plates without ZD01 were used as control. For the pathogenicity of different *A. solani* strains, 5-mm plugs of the wild type, Δ*slt2* and Δ*sod* mutants, and complementation strains were inoculated onto the center of one piece of fresh potato leaf. After 5 days of growth with 12 h of light and 12 h of darkness alternately at 25°C, the lesion areas were measured.

### SOD Concentration Measurement of *A. solani*

The concentrations of SOD in wild-type, Δ*sod* mutant, and complementation strains of *A. solani* were determined by double antibody sandwich method using a commercial kit. One gram of ground mycelium was resuspended in 9 ml of PBS buffer (pH 7.2–7.4) and then centrifuged at 3,000 rpm for 20 min. The supernatant was then collected for SOD concentration measurement using the Enzyme Immunoassay Kit for Superoxide Dismutase (Omega Bio-Tek, Norcross, GA, United States) according to the manufacturer’s instructions.

### Collection of VOCs With SPME and Analysis by GC-MS

For VOC extraction, the *Bacillus* strain was inoculated into 6 ml of LB medium in a 20-ml vial. After incubation at 37°C for 4 days, samples were used for analysis. To provide a repeatability of the experiment, four samples were prepared and the LB medium without the antagonistic bacteria was set up as a control.

Volatile organic compounds were analyzed using solid phase microextraction (SPME) coupled with gas-chromatography tandem mass spectrometry (GC-MS) analysis. The SPME fiber (2 cm, 50/30 μm divinylbenzene/carboxen/polydimethylsiloxane fiber, DVB/CAR/PDMS) was inserted into the headspace of the vial and then placed at 50°C for 40 min. Compounds were then desorbed for 10 min in the injection port of the gas chromatograph at 220°C with the purge valve off (split-less mode).

An HP-5 capillary column (30.0 m × 0.25 mm × 0.25 μm, Thermo) and helium as the carrier gas were used for GC-MS. A Thermo Trace 1300-ISQ MS was used for peak separation and detection. Each run was performed for 45 min. The initial oven temperature of 40°C was held for 4 min, ramped up at a rate of 5°C/min to 150°C holding for 1 min, further ramped up at a rate of 10°C/min to 280°C, and held for 5 min. The mass spectrometer was operated in the electron ionization mode at 70 eV with a source temperature of 280°C, and a continuous scan from 35 to 400 m/z was used. The analysis was performed in full-scan mode. Mass spectral data of the volatile compounds were compared with data in the National Institute of Standards and Technology (NIST) Mass Spectrum Library.

### Construction of Fungal Deletion and Complementation Strains

Gene deletion vector construction and transformation of *A. solani* were generated by the double-joint PCR method with minor modification (Yu et al., 2004). The primers used for flanking sequences amplification for each gene are listed in Supplementary Table S4. Open reading frames (ORFs) of *slt*2 and *sod* were replaced with a hygromycin resistance cassette (hyg) and the constructed fragment was inserted into the pEASY-T1 cloning vector (Supplementary Table S2). After transforming the constructed plasmid into HWC-168, the subsequent deletion mutants were verified by PCR with slt2-F/R and sod-F/R (Supplementary Table S4). For complementation, the respective ORFs were fused to a neomycin selection marker (neo) and introduced into the corresponding deletion mutants. Specific primers slt2-F/R and sod-F/R and marker gene primers neo-F/R (Supplementary Table S4) were used for verification.

### Quantitative Real-Time PCR

Total RNAs of *A. solani* cells co-cultured with volatiles produced by ZD01 after 3 and 4 days were extracted by using the Bacterial RNA Kit (Omega Bio-Tek, Norcross, GA, United States) according to the manufacturer’s instructions. First-strand cDNA was obtained using reverse transcriptase (TransGen Biotech, Beijing, China) with random hexamer primers. Real-time PCR was performed with SYBR Premix Ex Taq^TM^ (TransGen Biotech, Beijing, China). ITS gene was used as an internal reference gene. The specific primers used are listed in Supplementary Table S4. The relative expression of specific genes was calculated by using the 2^–ΔΔ*CT*^ method (Livak and Schmittgen, 2001).

### Statistical Analysis

Three independent experiments were performed for each assay. Data were analyzed by SPSS20.0 Windows Software (SPSS Inc., Chicago, IL, United States). Least significant differences (LSD) were calculated to compare the results at the 0.05 level.

## Data Availability Statement

The datasets generated for this study can be found in the online repositories. The names of the repository/repositories and accession number(s) can be found: https://www.ncbi.nlm.nih.gov/genbank/, CP046448.

## Author Contributions

DaZ, SY, YY, JinZ, and SF performed the experiments. DoZ and YP provided technical assistance. DaZ, ZY, and JieZ designed the experiments. DaZ, ZY, and JieZ wrote the manuscript. All authors contributed to the manuscript and approved the submitted version.

## Conflict of Interest

The authors declare that the research was conducted in the absence of any commercial or financial relationships that could be construed as a potential conflict of interest.
