# Supplementary material for: Antifungal Effects of Volatiles Produced by Bacillus subtilis Against Alternaria solani in Potato
Source: Front Microbiol. 2020 Jun 17;11:1196. doi: 10.3389/fmicb.2020.01196 (PMC7311636; doi:10.3389/fmicb.2020.01196)
Supplement: Supplementary file 1 [file Data_Sheet_1.doc]

**Table S1 Selection of** ***Bacillus* strains against *Alternaria solani* in rhizosphere soils from four different fields**

| **No.** | **Strains** | **Antifungal zone (mm)** | **Collection location** |
| --- | --- | --- | --- |
| 1 | Y18-7 | 10.50±0.61 | Chengde, Hebei |
| 2 | Y18-1 | 10.10±0.50 |
| 3 | Y18-4 | 9.98±0.79 |
| 4 | Y18-3 | 9.65±1.11 |
| 5 | Y18-11 | 9.63±1.31 |
| 6 | Y18-2 | 8.88±0.75 |
| 7 | Y18-23 | 8.75±0.90 |
| 8 | M18-3 | 8.50±0.58 |
| 9 | M18-5 | 8.35±0.37 |
| 10 | M18-15 | 8.30±0.95 |
| 11 | Y18-10 | 8.08±0.82 |
| 12 | X18-9 | 7.78±0.33 |
| 13 | Y18-15 | 7.65±0.47 |
| 14 | Y18-5 | 7.65±1.12 |
| 15 | X18-2 | 7.63±0.48 |
| 16 | M18-13 | 7.60±0.71 |
| 17 | Y18-6 | 10.33±0.90 | Qinhuangdao, Hebei |
| 18 | Y18-0 | 10.03±1.42 |
| 19 | Y18-16 | 9.50±1.15 |
| 20 | X18-10 | 8.83±0.56 |
| 21 | X18-1 | 8.48±0.41 |
| 22 | X18-9 | 8.15±0.51 |
| 23 | M18-5 | 8.10±0.35 |
| 24 | Y18-9 | 7.70±0.68 |
| 25 | X18-12 | 7.68±0.43 |
| 26 | X18-11 | 7.68±0.63 |
| 27 | X18-1 | 7.60±0.37 |
| 28 | Y18-13 | 7.60±1.25 |
| 29 | Y18-19 | 7.45±0.34 |
| 30 | X18-10 | 7.43±0.48 |
| 31 | HE-9 | 10.55±0.54 | Tengzhou, Shandong |
| 32 | HE-5 | 10.50± 0.59 |
| 33 | HE-7 | 10.18± 0.25 |
| 34 | HY-8 | 9.70± 0.39 |
| 35 | HE-8 | 9.53± 1.57 |
| 36 | HE-6 | 9.38± 0.55 |
| 37 | HY-18 | 9.35± 0.33 |
| 38 | HY-16 | 9.25± 0.87 |
| 39 | HY-9 | 9.18± 0.49 |
| 40 | HE-19 | 9.13± 0.59 |
| 41 | HE-17 | 9.13±0.33 |
| 42 | HE-15 | 9.03± 0.78 |
| 43 | HY-3 | 9.03± 0.67 |
| 44 | ZY-3 | 9.03± 0.17 |
| 45 | HE-12 | 9.00± 0.42 |
| 46 | HY-7 | 8.80± 0.66 |
| 47 | HE-07 | 8.75± 0.93 |
| 48 | ZY-8 | 8.70± 0.61 |
| 49 | HE-18 | 8.68± 0.81 |
| 50 | HE-16 | 8.60± 0.62 |
| 51 | WY-3 | 8.60± 0.59 |
| 52 | WY-8 | 8.45± 0.33 |
| 53 | HE-26 | 8.30± 0.80 |
| 54 | WY-4 | 8.23± 0.97 |
| 55 | HY-19 | 8.18± 0.97 |
| 56 | WY-9 | 8.10± 0.27 |
| 57 | HY-13 | 8.07± 0.67 |
| 58 | HE-28 | 8.00± 0.81 |
| 59 | WY-2 | 7.93± 0.31 |
| 60 | ZY-13 | 7.90± 0.80 |
| 61 | WY-6 | 7.88± 0.58 |
| 62 | HE-2 | 7.88± 0.74 |
| 63 | HE-3 | 7.80± 0.22 |
| 64 | HY-7 | 7.78± 0.79 |
| 65 | HY-3 | 7.68± 0.68 |
| 66 | WY-1 | 7.63± 0.41 |
| 67 | HE-29 | 7.60± 0.96 |
| 68 | HY-29 | 7.60 ±0.36 |
| 69 | WY-18 | 7.58± 0.31 |
| 70 | HE-10 | 7.58± 0.36 |
| 71 | ZD01 | 12.60±0.66 | Zhangjiakou, Hebei |
| 72 | ZJW-8 | 10.33±0.94 |
| 73 | ZJP-2 | 9.93±0.87 |
| 74 | ZJW-7 | 9.80±1.20 |
| 75 | ZJW-3 | 9.53±1.05 |
| 76 | ZJW-13 | 9.30±0.68 |
| 77 | ZJH-8 | 9.30±0.18 |
| 78 | ZJW-13 | 9.13±0.17 |
| 79 | ZJP-12 | 9.05±0.54 |
| 80 | ZJK-1 | 9.05±0.90 |
| 81 | ZJW-18 | 9.01±0.84 |
| 82 | ZJW-5 | 8.95±0.58 |
| 83 | ZJH-8 | 8.75±0.39 |
| 84 | ZJW-6 | 8.60±1.81 |
| 85 | ZJK-1 | 8.58±0.50 |
| 86 | ZJK-11 | 8.05±0.13 |
| 87 | ZJW-15 | 8.03±0.36 |
| 88 | ZJW-16 | 8.00±0.18 |
| 89 | ZJH-18 | 7.98±0.54 |
| 90 | ZJW-16 | 7.80±0.38 |
| 91 | ZJK-4 | 7.80±0.41 |
| 92 | ZJK-14 | 7.35±0.77 |
| 93 | ZJK-24 | 6.95±1.93 |
| 94 | ZJW-25 | 6.75±1.26 |
| 95 | ZJW-17 | 6.63±0.74 |
| 96 | ZJH-1 | 6.48±0.45 |
| 97 | ZJH-3 | 6.40±1.94 |
| 98 | ZJW-7 | 6.25±0.93 |
| 99 | ZJH-7 | 6.13±0.90 |
| 100 | ZJH-17 | 6.03±0.79 |
| 101 | ZJH-27 | 6.03±0.79 |
| 102 | ZJK-8 | 6.03±0.75 |
| 103 | ZJH-13 | 5.78±0.33 |

**Table S2 Strains and plasmids used in this study**

| **Strain and plasmid** | **Description** | **Source** |
| --- | --- | --- |
| **Strains** | | |
| *Bacillus subtilis* ZD01 | Undomesticated environmental strain | Lab strain collection |
| *Alternaria solani* HWC-168 | Undomesticated environmental strain | (Zhang, He et al. 2018) |
| HWC-168-01 | Δ*slt2*::hyg in HWC-168 (knock-out) | This study |
| HWC-168-02 | Δ*sod*::hyg in HWC-168 (knock-out) | This study |
| HWC-168-01C | Δ*slt*2::hyg::*slt*2::neo in HWC-168 (complementation) | This study |
| HWC-168-02C | Δ*sod*::hyg::*sodC*::neo in HWC-168(complementation) | This study |
| *Bipolaris sorokinianum* HA-B | Undomesticated environmental strain | Lab strain collection |
| *Rhizoctonia solani* HA-R | Undomesticated environmental strain | Lab strain collection |
| *Botrytis cinerea* HA-12 | Undomesticated environmental strain | Lab strain collection |
| *Aternaria mali roberts* HA-3 | Undomesticated environmental strain | Lab strain collection |
| *Alternaria alternata* B7 | Undomesticated environmental strain | Lab strain collection |
| *Fusarium solani* C13 | Undomesticated environmental strain | Lab strain collection |
| *Fusarium oxysporum* f.sp. *Vasinfectum* C5 | Undomesticated environmental strain | Lab strain collection |
| *Verticillium dahliae* *Kleb* C9 | Undomesticated environmental strain | Lab strain collection |
| *Fusarium graminearum* HA-9 | Undomesticated environmental strain | Lab strain collection |
| **Plasmid** | | |
| pEASY-T1 | Cloning vector | TransGen Biotech |

**Table S3 Chemical reagents used for inhibition test**

| **Chemical reagents** | **Source** |
| --- | --- |
| Dimethyl sulfoxide | Shanghai Macklin Biochemical Technology Co., Ltd |
| Acetophenone |
| Aniline |
| 2-Nonanone |
| m-Tolunitrile |
| 2-Ethylhexanol |
| 2-Heptanone |
| Benzylacetone |
| 4-Methylanisole |
| Diphenyl sulfide |
| p-Xylene |
| N-Acetylaniline |
| 1-Dodecanol |
| 2-Phenylacetophenone |
| Terephthalonitrile |
| 4-Methoxybenzyl alcohol |
| Diphenylamine |
| p-Anisidine |
| Benzothiazole |
| Valerophenone |
| 5-Methyl-2-hexanone |
| 2,5-Dimethylpyrazine |
| 2-Pentylfuran |
| 2-Naphthylamine | Shanghai Aladdin Biochemical Technology Co., Ltd |
| Benzoxazole |
| 6-Methyl-2-heptanone | Shanghai Xianding Biochemical Technology Co., Ltd |

**Table S4 Oligonucleotides used in this study**

| **Primer** | **Description** | **Sequence(5' →3' )** |
| --- | --- | --- |
| slt2-F | *slt*2 amplification and deletion verification | ATGGGCGACCTCGCCAACCGCA |
| slt2-R | *slt*2 amplification and deletion verification | TCATCGCATGCGACCATCAAGG |
| sod-F | *sod* amplification and deletion verification | ATGTGGGGCATATCGATGGTGTC |
| sod-R | *sod* amplification and deletion verification | TTAAATGGATGCCTTGAGCACGC |
| slt2-RT-F | *slt*2 qRT-PCR | CACTATTCAGGAACGCCAACC |
| slt2-RT-R | *slt*2 qRT-PCR | GACGACCTCAAACTGGAAATCAA |
| sod-RT-F | *sod* qRT-PCR | TCACGGATTATAGGCGTTGGA |
| sod-RT-R | *sod* qRT-PCR | CGGTGATGTCGGAGGTAGAAA |
| slt2-UP-F | *slt*2 upstream fragment amplification | GCACCGAATCGGATGACGAT |
| slt2-UP-R | *slt*2 upstream fragment amplification | GCCCAAAAAT GCTCCTTCAAGATGGCAGATGGCAGAGGCG |
| slt2-DOWN-F | *slt*2 downstream fragment amplification | CCCTGGGTTC GCAAAGATAATCGCAATCTGATAATGCTGTT |
| slt2-DOWN-R | *slt*2 downstream fragment amplification | TCCTCTGCTGCCTTCTCATCT |
| sod-UP-F | *sod* upstream fragment amplification | CCGCACACGTTTCTACCATGTT |
| sod-UP-R | *sod* upstream fragment amplification | GCCCAAAAAT GCTCCTTCAACTTGCCTGCCCCTCAAAGCAAT |
| sod-DOWN-F | *sod* downstream fragment amplification | CCCTGGGTTCGCAAAGATAAGTTATGGAAAACAATCTGAAAACGA |
| sod-DOWN-R | *sod* downstream fragment amplification | AGAATATTCATTAGTTCCATCCTAC |
| hyg-F | Amplify hygromycin resistance cassette | TCCGTCACCAACTCAACC |
| hyg-R | Amplify hygromycin resistance cassette | CAATAGCAGCCAGTCCCT |
| slt2-C-F | *slt*2 complementation | GAATTCGTCATTGGGATTGCTGTGGGAGGTG |
| slt2-C-R | *slt*2 complementation | CTCGAGTCATCGCATGCGACCATCAAGGC |
| sod-C-F | *sod* complementation | AAGCTTCTTGACGCTAGACGCGGATGCTTTC |
| sod-C-R | *sod* complementation | CTCGAGTTAAATGGATGCCTTGAGCACGCTA |
| neo-F | Amplify neomycin resistance cassette | TCCGTCACCAACTCAACC |
| neo-F | Amplify neomycin resistance cassette | CAATAGCAGCCAGTCCCT |

**REFERENCES**

Zhang, D., et al. (2018). "Genome sequence of the potato pathogenic fungus Alternaria solani HWC-168 reveals clues for its conidiation and virulence." **18**(1): 1-13.
